# Poor Law Topics

**Published:** 1906-04-28

**Authors:** 


### POOR LAW TOPICS.

### TOMMY ATKINS AND HIS PENSION.

There is often an outcry of indignant sympathy when some veteran of our army dies in the workhouse, but it cannot be denied that when a soldier comes to such straits he has, as a rule, only himself to blame, and his country has not been so ungrateful as is supposed. Many of these men are pensioners, and when quarter-day comes near they take their discharge, draw their pension, and squander it in a few days. Within a week they are back at the workhouse, penniless, and needing to be maintained out of the rates until the next instalment is due. This means that taxpayers are contributing twice over to maintain the same man. To prevent this injustice and control the thriftlessness that leads to it, a number of Boards of Guardians propose to ask the War Office to pay pensions weekly. This will certainly involve a great deal more work to that Office, and part of the money saved will have to be spent on the employment of extra staff there. Still, the matter is important, and even the pensioners themselves would benefit by being kept from wasting three months' maintenance in a few days. Failing this, one would suggest that Guardians should have a claim on an army pension, as they have on other funds belonging to paupers, for the cost of their maintenance. The knowledge that if he comes on the rates he has a chance of never fingering his pension at all would help some at least of our veterans to acquire the prudence and thrift which at present they lack.

